# Effects of Minimal Extracorporeal Circulation on the Systemic Inflammatory Response and the Need for Transfusion after Coronary Bypass Grafting Surgery

**DOI:** 10.1155/2019/1726150

**Published:** 2019-06-04

**Authors:** Mehmet Emre Elçi, Aydın Kahraman, Emre Mutlu, Cemil Selim İspir

**Affiliations:** ^1^Siyami Ersek Education and Research Hospital, Department of Cardiovascular Surgery, İstanbul, Turkey; ^2^The Ministry of Justice, Council of Forensic Medicine, İstanbul, Turkey; ^3^Marmara University, Faculty of Medicine, Cardiovascular Surgery, İstanbul, Turkey

## Abstract

**Objectives:**

The aim of this study is to compare the effects of the minimal extracorporeal circulation (MiECT) on postoperative systemic inflammatory response and the need for transfusion in patients undergoing open heart surgery with cardiopulmonary bypass.

**Methods:**

Patients were divided into two groups; Group M (*n*=31) included the patients operated via using the MiECT system and Group C (*n*=27) included the patients operated via using conventional cardiopulmonary bypass (CPB). Perioperative markers of inflammation after cardiopulmonary bypass in both groups were tested by measuring the levels via chemiluminescent immunometric assay. Blood samples were taken consecutively after anesthesia induction, 30^th^ minute of CPB, on the 6^th^, 24^th^, and 48^th^ hours after cardiopulmonary bypass.

**Results:**

The mean amount of priming solution was significantly lower in Group M when compared to Group C (802.60 ± 48.26 and 1603.71 ± 49.85 ml). The mean hematocrit (Hct) value taken immediately after cardiopulmonary bypass was found to be significantly higher in the MiECT patients with respect to the other group (% 32.71 ± 3.98 and % 28.82 ± 4.39). The transfused amounts of erythrocyte suspension and fresh frozen plasma were found to be significantly lower in patients in Group M when compared to those in Group C. Postoperative mediastinal drainage was also significantly lower in patients in Group M with respect to the other group. There was no significant difference between markers of inflammation.

**Conclusion:**

Our results show that MiECT seems to be more advantageous in terms of priming volume, perioperative hematocrit levels, need for blood and blood product transfusion, and mediastinal drainage with respect to the conventional approach after coronary artery bypass grafting.

## 1. Introduction

Cardiopulmonary bypass (CPB) is defined as a technique that temporarily replaces the function of the heart and lungs during cardiac surgery, maintaining the circulation of blood and the oxygen content of the body [1, 2]. The main principle of cardiopulmonary bypass is that venous blood is drained into the reservoir and passes through the oxygenator, through an arterial filter, and back into the patient.

Leukocytes, endothelial cells, and platelets are activated due to contact of the blood with foreign surfaces during cardiopulmonary bypass [3–6]. It is known that the systemic inflammatory response leads to postoperative morbidity and mortality. The resulting inflammatory response plays a primary role in the pathogenesis of cardiac, pulmonary, renal, hepatic, neurological, and hemostatic complications following cardiopulmonary bypass. The severity of inflammatory response during and after cardiopulmonary bypass can be reduced by increasing the biocompatibility of extracorporeal systems, by using filtration techniques, by using anti-inflammatory pharmacological agents, by using antioxidants, and by thermoregulation techniques.

In recent years, efforts have accelerated in order to reduce this inflammatory response. In this context, standard cardiopulmonary bypass systems have been modified to reduce the surface areas of extracorporeal circuits. Completely closed circuits have been developed to lower the volume of the prime solution for reducing hemodilution [7, 8] One of the newest technologies in this regard is CPB circuits called the minimal extracorporeal circulation system (MiECT) [9–11] Reducing the contact surface area and priming CPB systems with lower volumes have been shown to reduce the severity of the inflammatory response at certain rates [12, 13].

The minimal extracorporeal circulatory system consists of a centrifugal pump, a membrane oxygenator, a short heparin-coated line, and a vacuum line that can be added if necessary [14, 15]. The venous reservoir and the standard vacuum line in the conventional cardiopulmonary bypass system are not present in this circuit. Blood from the venous system does not accumulate in any area. It is a completely closed circuit that is not air related. This means that blood elements are exposed to less contact surface area and activity is kept at a lower level. The absence of a venous reservoir and the shorter length of the lines allow the prime volume used to be reduced as well.

The aim of our study was to compare the effects of minimal extracorporeal circulation system and conventional cardiopulmonary bypass circuits on postoperative transfusion requirement and inflammatory response. CRP, proinflammatory cytokines (IL-6, IL-8), TNF-*α*, and neutrophil elastase were used for assessing the inflammatory response.

## 2. Materials and Methods

In order to reduce the need for postcardiopulmonary bypass transfusion and the systemic inflammatory response in patients treated in the Department of Cardiovascular Surgery of Marmara University Faculty of Medicine, a single-blind, prospective, randomized trial using minimal extracorporeal circulation circuits was planned. The study was approved by the MUTF Research Ethics Committee (Protocol number: 09.2013.0218). All patients were informed in detail about the study. The consent form was signed by the patient and the researcher.

### 2.1. Patient Selection

The study included 58 consecutive patients who underwent cardiopulmonary bypass and isolated coronary artery bypass surgery between March and December 2013 by the Cardiovascular Surgery Clinic at Marmara University Faculty of Medicine. The MiECT system had an extra cost. Accordingly, we kept the number at 20. We did an equal number of control groups. Demographic data of the patients are given in Table 1.

Exclusion criteria: patients who read the information form but did not give consent, who underwent urgent surgery, who had a known malignancy, who had preoperative infection, who had low preoperative renal functions (preoperative serum creatinine level  > 1.2 mg/dl), who had preoperative liver disease (having abnormal liver function tests), who had preoperative ejection fraction < 40%, and who received preoperative intra-aortic balloon pump (IABP) support or inotropic drugs were not included in the study.

Because it was thought that interleukin-6,interleukin-8,TNF-alpha, and neutrophil elastase levels might be effective, patients who had any systemic infection and suspected systemic infection (white blood cell count ≥ 10,000, sedimentation rate ≥ 10 mm/h, and C-reactive protein level ≥ 5 mg/L) were excluded from the study. In addition, patients who received antibiotics within the last 15 days for any reason, who received steroids antibiotics within the last 15 days for any reason, who had unstable angina and preoperative myocardial injury (elevated levels of troponin and CK-MB), who underwent additional procedures due to intraoperative complications (heart injury, aortic dissection, vein injury, arterial injury, etc.), and who were under the age of 18 years were excluded from the study.

The patients were randomly divided into two groups: minimal extracorporeal circulation system (Group M, *n *:* *31) and conventional cardiopulmonary bypass system (Group C, *n *:* *27). Except for the cardiopulmonary bypass system used, the same anesthesia and surgical techniques were applied in both groups.

### 2.2. Cardiopulmonary Bypass and Surgical Technique

During cardiopulmonary bypass, perfusion was achieved with a roller pump (Sorin Group, Italy) in Group C and with a centrifugal pump (Maquet Jostra AG Group, Germany) in Group M. In Group C, hollow-fiber membrane oxygenator (DidecoR Compactflo Evo Phisio, Sorin Group, Italy) and arterial filter (DidecoR, Sorin Group, Italy) were used. In Group M, heparin-coated membrane oxygenator (Quadrox-i Adult, Jostra AG, Germany) was used. While 1/2 × 3/32 inch venous and 3/8 × 3/32 inch arterial polyvinyl chloride lines were used in Group C, 3/8 × 3/32 inch heparin-coated venous and arterial lines were used in Group M. The total prime volume was 1650 ml (1000 ml Isolyte S, 500 ml Gelofusine, and 150 ml Mannitol) in Group C and 800 ml (500 ml Isolyte S and 300 ml Gelofusine) in Group M, respectively. In this group, retrograde autologous priming was performed after cannulation, and this volume was taken back before CPB. Both groups also underwent nonpulsatile cardiopulmonary bypass. All patients received cefazolin sodium 1 g before performing an incision.

All operations were performed with standard median sternotomy. The left internal mammary artery (IMA) was used for revascularization of the left anterior descending artery (LAD). The great saphenous vein of appropriate length removed on the right or left lower extremity was used for revascularization of the other vessels. After the pericard was opened and suspended, 300 U/kg of heparin sodium was administered to Group C and 150 U/kg of heparin sodium was administered to Group M. An additional dose of heparin was administered so that the activated coagulation time was >400 seconds in Group C and between 250 and 300 seconds in Group M during CPB. The arterial flow was measured by aortic cannula (20-22-24 Fr according to the patient's BSA) (Cal MedR Lab, California, USA) placed in the ascending aorta. Because the diameters of the lines of the two systems placed in the right atrium of the patients were different, venous return was achieved with two-stage venous cannula (36/46–36/50 Fr) (Cal MedR Lab, California, USA) in Group C and with two-stage venous cannula (32/36–32/40 Fr) (Maquet, Jostra AG Group, Germany) in Group M. After the cannula was placed in the ascending aorta for cardioplegia and vent, CPB was performed. During the operations, while moderate systemic hypothermia (28–32°C) was used in Group C, superficial hypothermia (28–32°C) was used in Group M. After the clamp was placed in the ascending aorta, cardiac arrest was achieved with an antegrade delivery of 10 mL/kg of blood cardioplegia (plegisol + 30 meq potassium chloride + 10 meq sodium bicarbonate-1/4) at 5–7°C by applying a pressure of 120–140 mmHg in Group C and with an antegrade delivery of 10 mL/kg of blood cardioplegia (40 meq potassium chloride + 10 meq magnesium sulphate + 10 meq sodium bicarbonate) at the pump's heat by applying a pressure of 120–140 mmHg in Group M. Maintenance cardioplegia was delivered antegradely from the aortic root or grafts every 20 minutes.

The following points have been taken into consideration when deciding for blood transfusion in these patients: (1) the patient's anemia strengthening power, (2) the speed of ongoing bleeding, (3) the possibility of further blood loss, and (4) the risk of organ ischemia. Clinical parameters taken into consideration when deciding for blood transfusion are as follows: (1) age, (2) signs and symptoms of blood loss, (3) speed of blood loss, (4) cardiac function, (5) lung function, (6) ischemic heart disease, and (7) pharmacological treatment.

### 2.3. Follow-Up and Measurements

Arterial blood samples were collected from all patients after induction of anesthesia, 30 minutes after the onset of CPB, and 6, 24, and 48 hours after termination of CPB for measuring IL-6, IL-8, TNF-alpha, and neutrophil elastase.

IL-6, IL-8, TNF-alpha, and neutrophil elastase levels were measured using solid-phase, 2-site chemiluminescent immunometric assay commercial kits on the “Immulite 2000 XPi immunoassay system” device (Siemens Healthcare Diagnostic Products Ltd. Llanberis, Gwynedd LL55 4EL, United Kingdom) compatible with these kits. The CRP level was measured by the immunoturbidimetric method using commercial kits (CRPLX from Roche Diagnostics) on the “COBAS INTEGRA 800 analyzer” device (Roche Diagnostics GmbH, D-68298 Mannheim) compatible with these kits. All tests were made in accordance with the manufacturer's instructions.

### 2.4. Statistical Analysis

Mean, standard deviation, frequency, and ratio values were used for the descriptive statistics of the data. The distribution of the data was tested with the Kolmogorov–Smirnov test. The equality of the variants of the variables was tested. The *t*-test was used for the analysis of parametric data. The Mann–Whitney *U* test was used for the analysis of nonparametric data. The paired sample *t*-test was used for repeated measures. The chi-squared test was used for the analysis of proportional data. The Fisher's exact test was used when chi-squared test assumptions were not met. The SPSS 22.0 program was used for the analysis of the data.

## 3. Results

### 3.1. Perioperative Data of Patients

The perioperative data of the 58 patients included in the study are shown in Table 1. The mean number of bypass grafts was 3.25 ± 0.81 in Group C and 2.58 ± 0.71 in Group M, respectively (*p* > 0.05). The mean cross-clamp time was 35.74 ± 11.49 min in Group C and 36.41 ± 9.41 min in Group M, respectively. The mean duration of CPB was 84.92 ± 19.75 min in Group C and 85.55 ± 20.56 min in Group M, respectively. There was no statistically significant difference between the two groups in terms of mean cross-clamp time and mean duration of CPB (*p* > 0.05) (Table 1). The mean prime volume was 1603.71 ± 49.85 ml in Group C and 802.60 ± 48.26 ml in Group M, respectively. There was a statistically significant difference between the two groups in terms of mean prime volume (*p* < 0.05). The average amount of cardioplegic solution was 898.14 ± 169.37 ml in Group C and 558.06 ± 114.81 ml in Group M, respectively. There was a statistically significant difference between the two groups in terms of average amount of cardioplegic solution (*p* < 0.05). The mean body temperature during CPB was 31.59 ± 1.80˚C in Group C and 34.01 ± 0.042˚C in Group M, respectively. There was a statistically significant difference between the two groups in terms of mean body temperature (*p* < 0.05).

### 3.2. Postoperative Data of Patients

The postoperative parameters of the patients are shown in Table 2. The mean amount of drainage from chest tubes was 672.22 ± 157.09 ml in Group C and 446.77 ± 134.12 ml in Group M, respectively. There was a statistically significant difference between the two groups in terms of the mean amount of drainage from chest tubes (*p* < 0.05).

Although there was no statistically significant difference between the two groups in terms of hospital and intensive care unit length of stay, the mean duration of ventilation was 10.46 ± 1.83 hours in Group C and 6.21 ± 1.73 hours in Group M, respectively. There was a statistically significant difference between the two groups in terms of mean duration of ventilation (*p* < 0.05).

We evaluated perioperative blood transfusion rates. The mean perioperative erythrocyte suspension (ES) transfusion was 1.70 ± 0.66 U in Group C and 0.93 ± 0.89 U in Group M, respectively. There was a statistically significant difference between the two groups in terms of mean perioperative ES transfusion (*p* < 0.001). Similarly, the mean perioperative FFP transfusion was 2.51 ± 1.05 U in Group C and 0.93 ± 1.14 U in Group M, respectively. There was a statistically significant difference between the two groups in terms of mean perioperative FFP transfusion (*p* < 0.05). However, there was no statistically significant difference between the two groups in terms of mean perioperative platelet suspension (PS) transfusion (*p* > 0.05).

The preoperative and postoperative Hct values of the patients are shown in Table 3. There was no statistically significant difference between the two groups in terms of mean preoperative Hct value. The mean Hct value after leaving CPB was 28.82 ± 4.39 in Group C and 32.71 ± 3.98 in Group M, respectively. The mean Hct value at 24 hours postoperatively was 27.64 ± 4.27 in Group C and 31.9 ± 4.65 in Group M, respectively. There was a statistically significant difference between the two groups in terms of mean Hct value after leaving CPB and mean Hct value at 24 hours postoperatively (*p* < 0.05) (Table 4).

### 3.3. C-Reactive Protein Levels

There was no statistically significant difference between the two groups in terms of mean CRP values at 24 and 48 hours postoperatively (*p* > 0.05) (Table 5).

### 3.4. Interleukin-6 Levels

There was no statistically significant difference between the two groups in terms of mean IL-6 values before surgery, at 30 min of CPB, and at 6, 24, and 48 hours postoperatively (*p* > 0.05) (Table 6).

### 3.5. Interleukin-8 Levels

There was no statistically significant difference between the two groups in terms of mean IL-8 values before surgery, at 30 min of CPB, and at 6, 24, and 48 hours postoperatively (*p* > 0.05) (Table 7).

### 3.6. TNF-*α* Levels

There was no statistically significant difference between the two groups in terms of mean TNF-*α* levels (*p* > 0.05) (Table 8).

### 3.7. Neutrophil Elastase Levels

There was no statistically significant difference between the two groups in terms of mean neutrophil elastase levels values before surgery, at 30 min of CPB, and at 6, 24, and 48 hours postoperatively (*p* > 0.05) (Table 9).

## 4. Discussion

The heart-lung machine is an excellent device that makes the surgical treatment of many cardiac diseases possible nowadays. It temporarily takes over the function of the lungs and heart and maintains extracorporeal respiratory and circulatory support during this time. The proinflammatory cytokines become active due to contact of the blood with foreign surfaces during extracorporeal circulation [16]. The systemic inflammatory response after cardiopulmonary bypass may be limited to subclinical increase in levels of inflammatory mediators but may cause severe organ dysfunction or even death [17].

In our study, we observed that CRP, IL-6, IL-8, TNF-*α*, and neutrophil elastase levels increased rapidly after CPB in both groups. IL-6, IL-8, and neutrophil elastase levels peaked at 6 hours postoperatively, whereas TNF-*α* levels tended to continuously increase in consecutive measurements up to 24 hours postoperatively. CRP levels peaked at 48 hours postoperatively in consecutive measurements. In a study of Fromes et al. evaluating the inflammatory response after CPB as well as in a study of Ak et al. investigating lipoprotein lipase gene polymorphism and its effects on atherosclerosis, they found that IL-6 levels peaked at 6 hours after CPB. In this respect, we have obtained results consistent with the literature in our study [12, 18]. In a study of Delannoy et al. investigating sepsis after CPB and its association with postoperative CRP and procalcitonin levels, they observed that CRP levels peaked at 24 and 48 hours after CPB [19]. This finding was similar to the CRP data we obtained in our study. In a study of Alataş et al. evaluating myocardial ischemia reperfusion injury after CPB, they showed that TNF-*α* levels tended to increase even at 24 hours postoperatively. This result was also similar to our study [20, 21].

When the conventional cardiopulmonary bypass and MİECT groups were compared with each other, there was no statistically significant difference between the two groups in terms of mean IL-6, IL-8, TNF-*α*, and neutrophil elastase levels at all postoperative sampling times (*p* > 0.05).

The fact that the inflammatory parameters were similar for the two groups in our study can be explained by the fact that hypothermia was administered at a higher rate during cardiopulmonary bypass in the conventional group. Studies have shown that hypothermia is protective against the inflammatory response. Another explanation in this regard can be that cardiopulmonary bypass time is short in both groups and the inflammatory response becomes obvious, and consequently, the protective effect of MİECT does not become evident.

When we examined the effects of hypothermia on leukocyte activation, cytokine balance, and thus postoperative organ damage, Quing et al. reported that hypothermia may be beneficial in organ preservation by suppressing TNF-*α* production during cardiopulmonary bypass [22]. This idea is also supported by the fact that Menasche et al. have suggested that hypothermia administered during CPB reduces the inflammatory response with low cytokine production [23–25].

We think that the anti-inflammatory effect may become more prominent with deepening hypothermia in patients operated on with the minimal extracorporeal circulation system. On the other hand, reducing the hypothermia protocol applied during CPB to lower temperatures in centers using the minimal extracorporeal circulation system may also be effective in observing the suppressive effect of hypothermia on the inflammatory response.

In recent years, many studies have reported that hemodilutional anemia, which occurs during CPB, causes organ dysfunction and increases morbidity and mortality [26, 27]. In our study, the mean Hct value after leaving CPB was higher in patients operated on with the MİECT system. These patients had lower blood transfusion rates. It is known that blood transfusion during coronary bypass surgery reduces long-term survival rates [28, 29]. These data continue to push perfusion technology to design circuits which allow use of a lower prime volume and have less contact surface area. In our study, the groups operated using the minimal extracorporeal circulation system and conventional cardiopulmonary bypass system were compared with each other in terms of the need for erythrocyte suspension and fresh frozen plasma transfusion and total length of stay. We observed a statistically significant decrease in these variables in the MiECT group (*p* < 0.05). In this context, minimal extracorporeal circulation has recently become popular. The fact that studies have found that markers of systemic inflammatory response are lower in patients operated on with the MİECT system has suggested that it would bring advantages in terms of mortality and morbidity. However, the long-term results of the use of the MiECT system in terms of morbidity and mortality have not yet been fully demonstrated [30, 31]. The reasons such as the fact that the MiECT system requires a certain learning process, has a risk of perioperative venous air leakage, creates a concern for microemboli formation, and restricts surgical field aspiration limit its use. In addition, the fact that open heart operations can be performed optimally and easily with acceptable mortality and morbidity rates by conventional methods nowadays reveals the more widespread use of conventional methods. Geratti et al. found that the MİECT system significantly increased the postoperative Hct values and significantly decreased the blood transfusion requirements [31, 32]. In a study of Stadler et al., they reported that blood transfusion rates were similarly reduced by the prevention of intraoperative hemodilution [33]. In a study of Severdija et al. using the retrograde autologous priming method in standard cardiopulmonary bypass systems, the retrograde autologous priming method yielded a nearly twofold gain in prime volume. This gain in the same study has been shown to increase intraoperative Hct levels and to reduce the need for blood transfusion in the patients [34]. In a study conducted by Ohata et al., hematocrit values were assessed during and after cardiopulmonary bypass. They showed that hematocrit values were significantly higher in the group using the MİECT circuit [35].

In our study, we evaluated the effect of modified cardiopulmonary bypass system (i.e.,use of minimal extracorporeal circulation system) on postoperative transfusion requirement and systemic inflammatory response. In our study, the molecules (IL-6, IL-8, TNF-*α*, neutrophil elastase, and CRP), which we think to be indicative of the severity of the systemic inflammatory response, were used in patients who had similar demographic characteristics and had similar cross-clamp time and CPB duration. In our study, it was observed that the mean blood plasma concentrations of these molecules in the samples taken at different times preoperatively and postoperatively were significantly increased after CPB. When the mean postoperative values of these molecules were compared between the two groups, there was no statistically significant difference in both groups except for IL-6 value at 6 hours postoperatively, TNF-*α* value at 48 hours postoperatively, and neutrophil elastase value at 30 min of CPB. The fact that the mean postoperative values of IL-6, IL-8, TNF-*α*, neutrophil elastase, and CRP did not differ significantly between the two groups despite the application of different hypothermia protocols in the two groups at all sampling times promises possible gains for the future. The most significant difference found in our study was that the prime volume and blood-surface contact area were reduced with the use of minimal extracorporeal circulation system. This difference significantly increased the intraoperative and postoperative Hct values as well as significantly reduced the need for postoperative erythrocyte suspension and fresh frozen plasma transfusion in the group using the minimal extracorporeal circulation system.

Our study had some weak points. It was conducted in a small number of patients and in a single center. The surgical interventions were compatible because all patients were operated by the same surgical team and the same anesthesia team. However, there were a small number of patients. Retrograde autologous priming could not be made in the group using conventional CPB system in accordance with routine practice in our clinic. The cardioplegia protocol differed between the two groups. The number of patients participating in the study was not sufficient to examine the cause-effect relationship for postoperative complications such as chronic renal failure (CRF), cerebrovascular event, myocardial infarction, and mortality. The study included relatively low-risk patients who had normal preoperative ejection fraction, who had normal preoperative kidney and liver functions, and who had no chronic disease and were negative for inflammatory markers. From the fact that open heart operations can be performed with low morbidity and mortality rates by standard methods in a group of low-risk patients, it can be considered that more valuable gains can be obtained in a group of high-risk patients. In this context, it can be considered that this study should also be performed in a group of high-risk patients, in long-term and more complex operations, in patients with chronic renal failure, or in patients with preoperative left ventricular dysfunction.

As a result, our study demonstrated that minimal extracorporeal circulation system allowed use of a lower prime volume, reducing intraoperative hemodilution and the need for postoperative blood transfusion. However, we found that there was no statistically significant difference between minimal extracorporeal circulation system and conventional cardiopulmonary bypass system in terms of inflammatory response. Minimal extracorporeal circulation systems can bring advantages in the reduction of systemic inflammatory response by establishing lower temperatures during cardiopulmonary bypass and by utilizing the positive effects of hypothermia on the inflammatory response.

## Figures and Tables

**Table 1:** Baseline characteristics of the patient groups.

			Group C	Group M	*P*
Age		(mean ± SD)	60.25 ± 10.08	63.29 ± 8.52	*P*=0.61
Sex	Female	(*n*/%)	6 ± 22.20%	4 ± 12.90%	*P*=0.56
	Male	(*n*/%)	21 ± 77.80%	27 ± 87.10%	
Weight (kg)		(mean ± SD)	167.14 ± 5.70	171.03 ± 6.65	*P*=0.45
Height (cm)		(mean ± SD)	73.44 ± 11.20	81.58 ± 11.31	*P*=0.77
EF%		(mean ± SD)	54.37 ± 9.19	56.51 ± 8.75	*P*=0.35
Euroscore II%		(mean ± SD)	1.72 ± 1.01	1.65 ± 0.82	*P*=0.49
CCS	1^*∗*^2	(*n*/%)	23 ± 83.7%	26 ± 81.8%	*P*=0.67
	3^*∗*^4	(*n*/%)	4 ± 17.3%	5 ± 19.2%	
NYHA	1^*∗*^2	(*n*/%)	24 ± 88.9%	27 ± 87.1%	*P*=0.29
	3^*∗*^4	(*n*/%)	3 ± 11.1%	4 ± 12.9%	
DM		(*n*/%)	15 ± 55.6%	15 ± 48.4%	*P*=0.15
HT		(*n*/%)	24 ± 88.9%	25 ± 80.6%	*P*=0.38
Hyperlipidemia		(*n*/%)	12 ± 44.4%	10 ± 33.2%	*P*=0.25
Smoking		(*n*/%)	20 ± 74.1%	22 ± 71%	*P*=0.75
Alcohol		(*n*/%)	1 ± 3.2%	1 ± 3.7%	*P*=0.54

CCS: Canadian Cardiovascular Society; NYHA: New York Heart Association classification; CPB: cardiopulmonary bypass; EF: ejection fraction; DM: diabetes mellitus; HT: hypertension; SD: standard deviation.

**Table 2:** Perioperative data of the patients.

		Group C	Group M	*P*
Previous CABG number	(mean ± SD)	3.25 ± 0.81	2.58 ± 0.71	*p*=0.27
Duration of operation (min)	(mean ± SD)	262.22 ± 44.49	240.64 ± 40.16	*p*=0.16
Duration of CPB (min)	(mean ± SD)	84.92 ± 19.75	85.85 ± 20.56	*p*=0.17
Cross-clamp time(min)	(mean ± SD)	35.74 ± 11.49	36.41 ± 9.41	*p*=0.13
Prime (cc)	(mean ± SD)	1603.71 ± 49.85	802.60 ± 48.26	*p*=0.0001
Cardioplegia (cc)	(mean ± SD)	898.14 ± 169.37	558.06 ± 114.81	*p*=0.0001
Hypothermia (˚C)	(mean ± SD)	31.59 ± 1.80	34.01 ± 0.042	*p*=0.02

CABG: coronary artery bypass grafting; CPB: cardiopulmonary bypass.

**Table 3:** Postoperative data of the patients.

		Group C	Group M	*P*
Length of hospital stay (day)	(mean ± SD)	6.59 ± 1.42	7.09 ± 1.79	*P*=0.45
Ventilation time (hour)	(mean ± SD)	10.46 ± 1.83	6.21 ± 1.73	*P*=0.0001
ICU stay (hour)	(mean ± SD)	41.77 ± 21.66	34.83 ± 18.42	*P*=0.25
Drainage volume (cc)	(mean ± SD)	672.22 ± 157.09	446.77 ± 134.12	*P*=0.0001
İnotrope use	(*n*/%)	11 ± 40.7%	12 ± 38.7%	*P*=0.15
Red blood cell transfusion (U)	(mean.±SD)	1.70 ± 0.66	0.93 ± 0.89	*P*=0.0001
Fresh frozen plasma transfusion (U)	(mean ± SD)	2.51 ± 1.05	0.93 ± 0.20	*P*=0.0001
Platelet transfusion (ml)	(mean ± SD)	0.23 ± 0.10	0.22 ± 0.10	*P*=0.35

ICU: intensive care unit.

**Table 4:** Biochemical values of the patients.

		Group C	Group M	*P*
Hematocrit	Preop	37.11 ± 4.60	37.64 ± 5.46	*P*=0.64
	POMP OUTPUT	28.82 ± 4.39	32.71 ± 3.98	*P*=0.18
	Postop-24^th^ hour	27.64 ± 4.27	31.9 ± 4.65	*P*=0.001

Troponin	Preop	36.29 ± 9.51	26.92 ± 5.71	*P*=0.12
	POMP OUTPUT	323.96 ± 84.81	241.88 ± 61.42	*P*=0.53
	Postop-24^th^ hour	789.61 ± 235.92	427.55 ± 261.29	*P*=0.001

Creatine kinase	Preop	67.62 ± 32.51	92.38 ± 52.66	*P*=0.22
	POMP OUTPUT	317.22 ± 107.74	335.61 ± 231.98	*P*=0.26
	Postop-24^th^ hour	679.77 ± 403.16	579.06 ± 467.60	*P*=0.48

CK-MB	Preop	2.03 ± 1.08	2.25 ± 1.90	*P*=0.61
	POMP OUTPUT	22.05 ± 7.56	17.42 ± 8.02	*P*=0.001
	Postop-24^th^ hour	30.86 ± 9.33	20.09 ± 5.29	*P*=0.74

**Table 5:** C-reactive protein levels.

		Group C	Group M	*P*
CRP (C-reactive protein)	Preop	3.25 ± 3.01	2.64 ± 1.70	*P* = 0.73
	End-CBP	2.54 ± 2.43	3.46 ± 2.66	*P* = 0.63
	Postop-24^th^ hour	64.49 ± 20.24	78.63 ± 40.45	*P* = 0.1
	Postop-48^th^ hour	239.22 ± 46.05	215.20 ± 79.59	*P* = 0.25

**Table 6:** Levels of IL-6.

		Group C	Group M	*P*
IL-6 (pg/ml)	Preop	1.69 ± 3.74	0.81 ± 1.37	*P*=0.47
	CPB 30^th^ minute	2.83 ± 5.06	1.99 ± 4.86	*P*=0.84
	Postop-6^th^ hour	34.70 ± 15.22	32.50 ± 18.04	*P*=0.74
	Postop-24^th^ hour	18.19 ± 22.43	17.20 ± 7.88	*P*=0.68
	Postop-48^th^ hour	6.69 ± 8.36	9.59 ± 7.64	*P*=0.38

**Table 7:** Levels of IL-8.

		Group C	Group M	*P*
IL-8 (pg/ml)	Preop	21.37 ± 26.32	22.54 ± 12.82	*P*=0.65
	CPB 30^th^ minute	24.55 ± 21.73	22.80 ± 17.11	*P*=0.42
	Postop-6^th^ hour	30.70 ± 32.35	30.00 ± 20.91	*P*=0.36
	Postop-24^th^ hour	23.14 ± 21.55	22.58 ± 14.00	*P*=0.55
	Postop-48^th^ hour	22.03 ± 16.96	22.29 ± 22.85	*P*=0.76

**Table 8:** Levels of TNF-*α*.

		Group C	Group M	*P*
TNF-*α*(pg/ml)	Preop	13.79 ± 4.79	13.42 ± 0.84	*P*=0.74
	CPB 30^th^ minute	14.17 ± 5.71	13.90 ± 9.32	*P*=0.66
	Postop-6^th^ hour	14.32 ± 4.33	13.98 ± 2.12	*P*=0.28
	Postop-24^th^ hour	15.12 ± 9.30	14.73 ± 4.17	*P*=0.17
	Postop-48^th^ hour	12.43 ± 4.18	13.87 ± 7.49	*P*=0.49

**Table 9:** Levels of neutrophil elastase.

		Group C	Group M	*P*
Neutrophil elastase (pg/ml)	Preop	81.4 ± 23.34	83.48 ± 46.08	*P*=0.35
	CPB 30^th^ minute	104.29 ± 40.21	108.12 ± 66.54	*P*=0.88
	Postop-6^th^ hour	137.85 ± 97.16	152.96 ± 136.33	*P*=0.56
	Postop-24^th^ hour	97.88 ± 36.42	107.93 ± 49.70	*P*=0.75
	Postop-48^th^ hour	80.77 ± 40.29	81.51 ± 59.54	*P*=0.22

## Data Availability

The data used to support the findings of this study are available from the corresponding author upon request.
